# Sociocultural practices and COVID-19 prevention: A qualitative study of Mtwara, Shinyanga, and Arusha, Tanzania

**DOI:** 10.4102/jphia.v16i3.713

**Published:** 2025-04-18

**Authors:** Egidius Kamanyi, Magolanga Shagembe, Richard Sambaiga, Chima E. Onuekwe, Tumaini Haonga, Ambrose T. Kessy, William Mwengee

**Affiliations:** 1 Department of Sociology and Anthropology, College of Social Sciences, University of Dar es Salaam, Dar es Salaam, United Republic of Tanzania; 2 Tanzanian Psychological Association (TAPA), Dar es Selaam, United Republic of Tanzania; 3 Department of Immunizations, Emergency Preparedness and Response (EPR), World Health Organization, Dar es Salaam, United Republic of Tanzania; 4 Centre for Health and Allied Legal and Demographical Development, Research and Training (CHALADDRAT), Nnamdi Azikiwe University, Awka, Anambra State, Nigeria; 5 Department of Health Promotion Unit, Ministry of Health, Dodoma, United Republic of Tanzania; 6 Directorate of Research, Publications and Consultancy, University of Dodoma, Dodoma, United Republic of Tanzania; 7 Department of Planning, Finance and Administration, The Law School of Tanzania, Dar es Salaam, United Republic of Tanzania

**Keywords:** COVID-19, public health, preventive measures, sociocultural practices, vaccine hesitancy, health interventions, community response

## Abstract

**Background:**

The World Health Organization pronounced COVID-19 as a public health emergency in March 2020. Studies conducted in Tanzania and beyond indicate that poor literacy, limited understanding of the disease, challenging living conditions, increasing poverty, and unemployment are key determinants, while the influence of sociocultural factors has received less attention. This study reinforces the position of sociocultural practices in determining how people practiced the preventive measures against COVID-19 pandemic.

**Aim:**

This qualitative study explores the influence of sociocultural practices in the implementation of COVID-19 preventive measures in Arusha, Mtwara and Shinyanga regions of Tanzania.

**Setting:**

This study was conducted in Tanzania, covering diverse sociocultural contexts of Mtwara, Arusha and Shinyanga regions.

**Methods:**

Focus group discussions, key informant interviews and rapid ethnographic field observations were used to obtain more detailed information from study participants.

**Results:**

Findings suggest that sociocultural practices shaped how individuals and communities responded to COVID-19 preventive measures, influencing acceptance, hesitation, resistance, or modification of public health guidelines, including vaccine uptake. In Mtwara, Arusha, and Shinyanga, people embraced their sociocultural practices to navigate the new disease, whose origins were debated or unknown. Their responses to the pandemic were mediated by sociocultural practices and other factors.

**Conclusion:**

Sociocultural practices shaped the acceptance, adaptation, or resistance to COVID-19 measures in Tanzania, emphasising the need for community-integrated public health strategies.

**Contribution:**

This study underscores the impact of sociocultural factors on public health, offering insights for socioculturally tailored pandemic interventions.

## Introduction

Local contexts have profoundly shaped the way various communities across the world responded to the public health preventive measures against COVID-19 pandemic. In Tanzania, sociocultural practices play a significant role in shaping community behaviour, particularly in response to health interventions. This is because Tanzanian society highly values communal living and social gatherings, which are central to its cultural fabric. These cultural practices not only define and set the social norms by which the community is guided but also influence how interventions are received and internalised. This strong sense of community, while vital to the Tanzanian way of life, may have posed challenges to the adoption of COVID-19 preventive measures such as social distancing and mask-wearing. Despite government efforts to enforce these measures, resistance was observed, likely because of the tension between public health directives and deeply ingrained sociocultural practices. Traditional beliefs, communal bonds and reliance on home remedies may have further influenced the public’s response, complicating the implementation of preventive strategies from World Health Organization (WHO), responsible health ministries and other duty bearers in the health sector. However, the role played by these sociocultural practices in determining the success or failure of COVID-19 prevention efforts in Tanzania remains unclear. Understanding this role is critical to informing future public health interventions, particularly in contexts where cultural norms may conflict with recommended health practices. Therefore, this study seeks to explore the influence of sociocultural practices on the implementation of COVID-19 preventive measures in Tanzania, aiming to fill a gap in the current literature and provide insights for improving health interventions in culturally diverse settings focussing on a qualitative study in Mtwara, Arusha and Shinyanga regions.

### Study context

COVID-19 is marked as one of the pandemics to have ever devastated the world’s entire populace. Its emergence in 2019 was received with mixed views, beliefs, expectations and reactions. Its spread was sporadic and casualties, most of whom succumbed to rapid death, added to the dilemma and trauma across the public health sector and the scientific community.^1,2^ The WHO pronounced it as a public health emergency (PHE) on 11 March 2020.^3^ While COVID-19 is not the first PHE, the way it overwhelmed the public in general and the health sector in particular remains a matter of higher attention. Unlike severe acute respiratory syndrome (SARS), which caused 800 deaths in 8000 cases in 2002; Swine flu (H1N1) in 2009 with 18 500 deaths; Middle East respiratory syndrome (MERS) in 2012 that affected 2500 with 800 deaths; and Ebola in 2014 with 28 616 cases and 11 310 deaths, the COVID-19 pandemic has recorded more than 730 000 cases with at least 35 000 deaths. Several factors are responsible for such catastrophic loss of human life such as unemployment, education and democratisation.^4,5,6^ And as argued by Obasanjo et al.,^4^ low education levels and high unemployment were associated with higher death rates from COVID-19, yet they believed ‘… it is not well understood what factors modulated the rate of COVID-19 cases and death on the continent’.^4^ Other factors mentioned in the literature include population density, aged and vulnerable populations, inefficient health system capacity, government response, travel and international connections, existing healthcare disparities, economic and social factors, poor vaccine uptake and misinformation.^7,8,9,10,11^

Selvitella and Foster,^12^ argue that social factors impeding vaccine uptake and discouraging other public health measures include preference for traditional and home remedies and influence from others. While this is correct, the influence of sociocultural practices in the context of Arusha, Mtwara and Shinyanga is unknown. Societal belief systems that are eminent in their sociocultural practices have proven to be at the centre of the success or failure of several health and other community interventions,^13,14,15,16,17^ but their influence in the case of COVID-19 prevention is not well-established in literature. Establishing how these factors played out during COVID-19 prevention adds significantly to the existing knowledge gap by responding to the lack of clarity about the subject in study areas, especially considering that Tanzania has a rich cultural identity and adherence to their cultural values linked to age, gender, medication and administrative system could influence the COVID-19 preventive measures.

Hence, in this article, we particularly, explore the influence of sociocultural practices on the implementation of COVID-19 preventive measures in Mtwara, Arusha and Shinyanga regions of Tanzania.

## Research methods and design

This qualitative study predominantly applied focus group discussions (FGDs) and key informant interviews (KIIs) triangulated with secondary data from relevant documents as main data generation methods. Focus group discussions and KIIs were conducted and/or moderated by facilitators supported by assistants. All facilitators met all participants prior to the commencement of study data collection to provide them with basic information about the aims of the study, including explaining issues of social ethics such as administering informed consent after explaining the intention and expected benefits of the study and ensuring their confidentiality. The study as a whole was conducted in eight regions of the Tanzanian mainland. In each region, 8 FGDs and 120 KIIs were conducted. Thus, a total of 24 FGDs with at least 288 participants having attended the discussions, and 45 KIIs were conducted with key informants, with 15 of them coming from each region. Each FGD had a minimum of 12 participants. Key informant interviews were conducted with community leaders and members. Focus group discussions and KIIs conducted in Swahili were transcribed and translated into English, and later analysed using NVivo version 12 software. Data from Mtwara, Shinyanga, and Arusha in districts of Mtwara District Council (DC), Nanyamba Town Council (TC), and Mtwara Municipal Council (MC); Kishapu DC, Shinyanga DC, and Shinyanga MC; Meru DC, Arusha City Council (CC), Arusha DC and Karatu DC, respectively, have been used in this article.

### Ethical considerations

Ethical approval to conduct this study was obtained from the University of Dodoma Research Review Ethics Committee (No. MA.84/261/76/214).

## Results

### Gendered decision-making powers vested in men

All participants in KIIs and FGDs believe that men have a major say regarding decisions about COVID-19 preventive measures. It was reiterated that men because of the patriarchal system, always maintain the role of making decisions concerning the welfare of the family, including socioeconomic matters, particularly health decisions. The study showed that men decided whether family members could observe social distancing, avoid gathering, use face masks, apply hand washing and uptake vaccines. In one FGD, participants had a consensus on the following narrative:

‘I think it has to be understood that our husbands and community leaders, who are mostly men, had much to play in the way other people, especially women, and children, acted towards preventive measures against COVID-19; it is men who make most of the decisions in the community. If your husband or father decides not to buy the required masks or prohibits you from going to the hospital to be vaccinated, you have no choice but to follow.’ (FGD 20, P10, Community member, Shinyanga)

Many participants agreed with these points of view. They considered the level of education, economic status and residence to be some of the factors that influenced these unequal gendered relations, meaning that some of the educated men or those economically well-off and those living or exposed to urban life were more likely to devolve decision-making power to their wives. They could also accept other members of the family adopting the conventional preventive behaviours for the COVID-19 pandemic that was in place. Although the study indicates that most men were the central decision-makers towards adherence or non-adherence to the best practices against the pandemic.

### Age-set organisation and respect for the elderly

We found that age is regarded as an important aspect with regard to knowledge, wisdom and awareness, and gives power to people in the cultural hierarchy. For instance, the study found that among the Maasai of Arusha and Sukuma in Shinyanga, people looked up to what their elderly had to say about the COVID-19 pandemic including its origin, symptoms, preventive measures and consequences. Study participants indicated their belief in what older people would trust as the right course of action. This fact was not peculiar to COVID-19 preventive measures alone but rather something longstanding in society. Age was seen as a very important asset in making the right decisions towards desirable health-seeking behaviours. As one of the local leaders narrated:

‘I can assure you that the role of the elderly in influencing the decisions made by our community members was of high level; people would hear most news on the media, at health facilities, from peers and other sources but the most trusted source to them was what their elderly said and accepted as the right thing to do.’ (KII 18, Local leader, Mtwara)

We further found that not only did the elderly set the grounds for what the community could accept as the right decision, but they also created narratives that helped to reduce the level of anxiety and trauma because of devastating news about the severity of the disease and its fatal potential elsewhere. One of the participants stated the following:

‘In this community, elderly people have been important in many things, particularly in controlling the panic against COVID-19; stating that this disease is not the first complicated to ever happen and providing part examples gave a lot of hope to people and many believed them.’ (FGD 8, P6, Community member, Arusha)

It was evident that the elderly, who are also regarded as the traditional experts, influenced the way community members responded to the COVID-19 pandemic in terms of following or disregarding the preventive measures in place. It was also found that the role of the elders in determining decisions about vaccine uptake was crucial and has remained critical. Furthermore, evidence from the field attests to the fact the elderly acted as communicators of what is right or wrong to do during the pandemic.

### Collective work ethic among communities

While working collectively was perceived by many participants as a practice of great value to their communities, it also has a limitation to adhering to certain preventive measures against the COVID-19 pandemic. In particular, non-pharmaceutical interventions (NPIs), such as lockdowns and social and/or physical distancing were contravened by this sociocultural practice among the community members as evident in the following narrative from a healthcare worker in Shinyanga:

‘Sukuma people who mainly farmers and pastoralists strongly believe in working in groups … telling them to stay a distance or every family staying at home meant diverting their normal ways of working … it was difficult and near to impossible to convince them otherwise.’ (KII 35, Health worker, Shinyanga)

This above-stated view was also supported in other FGDs:

‘We want to assure you that we refused to stop our working traditions, we cannot fall to the trick of dividing us, we know people in urban areas have adopted the West culture of individualism and non-collaboration, let them proceed … and as you can see, we are still alive.’ (FGD 17, P3, Community member, Shinyanga)

Findings indicate that this was common in all the areas wherein we conducted this study. Although with varying intensity and insistence on the value of this practice, where it was strongest among the Sukuma in Shinyanga, the same was observed in FGDs and KIIs conducted in Arusha and Mtwara. Participants believed that corresponding to the protocols prohibiting them from working together was a way to make them poor and they insisted this could lead to everlasting social and cultural disturbances.

### Meaningful cultural gatherings and the existence of extended families

As previously shown in this article, dynamism in practices of social behaviours such as care for patients, social gatherings, traditional care systems and appraisal of norms versus biomedical demands such as public health and social measures (PHSMs) has remained rife in the understanding of how communities interacted with COVID-19 in Tanzania in general and in Mtwara, Arusha, and Shinyanga in particular. Cultural gatherings of different purposes and living in extended family setups were found as among the sociocultural practices of critical significance for communities in Tanzania. We observed that there is a myriad of such cultural gatherings in these communities. Some involve culturally significant *rites of passage*. We further noticed in discussions with participants that many communities in Mtwara, Arusha and Shinyanga have initiation ceremonies that mark the transition from childhood to adulthood. Such ceremonies often involve a series of rituals, such as circumcision for boys or other forms of initiation for girls. These are regarded as an essential part of these ceremonies, allowing family and community members to meet to celebrate the transition and offer support to the initiates. One participant mentioned the following during an interview:

‘For us in Mtwara, ‘Unyago na Jando’ [*the initiation ceremony for girls and boys, respectively*] is very important because every year there is a cohort of boys and girls that need to be initiated into the next stage of their lives, which if not done, impedes what they can do next … thus, without a shocking incidence from COVID-19 people didn’t trust they were in danger, hence carried on with their cultural gatherings.’ (KII 25, Elderly, Mtwara)

The same practices were also mentioned among the communities in Arusha and Shinyanga among the Maasai. For example, the same initiation practice involves the young boys turning to Moran (young Maasai men forming warrior groups), who are a group of community security forces for people and herds, while for the Sukuma, the practice allows boys and girls at puberty to meet and exchange teachings with elders and be able to select partners commonly referred to as ‘*chaghoulagha*’ [select or choose one]. These ceremonies also involve the showcasing of energetic traditional dances that people enjoy. For that reason, while there were indications that there were families that avoided such gatherings, the majority still proceeded with the practice, notwithstanding the available preventive measures. Other important gatherings mentioned included marriage, burial or funeral, naming ceremonies and harvest festivals in various ethnic groups, including Mtwara, Arusha, and Shinyanga. At these ceremonies and gatherings, handshaking, embracing and hugging are part of the practice that are signs of sharing joy and happiness or sadness and sorrow, and the same are highly valued.

### Existence of strong traditional administrative systems

Beyond the aforementioned respect for elderly people, the study established that there is strong traditional leadership, especially among the Sukuma and Maasai of Shinyanga and Arusha, respectively. For instance, the presence of ‘*Watemi*’ [chiefs] and ‘*makamanda*’ [commanders], who have the highest power in any decision-making process in the community, did impact how people responded to preventive measures implemented against the COVID-19 pandemic, including the dynamics of vaccine hesitancy and acceptance. These traditional administrative systems work along with the formal governance system, yet people expressed a stronger sense of trust in the former. For instance, we noticed that it is the Laigwanan or Laibon who form the elder council, which is a powerful traditional body for conflict resolution and decision-making. The presence of such leaders who practice sociocultural codes of behaviours and practices meant other community members depended on these people’s wisdom to trust and abide by the preventive measures. A health worker had the following views:

‘The traditional administrative leaders have had a major influence on how communities responded to COVID-19 diseases. I am aware that among the Maasai, all decisions that affect their community are to be made by the Laigwanan; without their consent, no new practices can be accepted.’ (KII 8, Health worker, Arusha)

This view was corroborated in other areas, including Shinyanga and Mtwara. In Shinyanga, for instance, it was found out that the traditional leaders were in support of Magufuli, the late President of Tanzania in power between 2016 and 2021, who had opposing views to COVID-19 pandemic public health measures, especially lockdowns, and vaccines, believing it was a hoax (https://www.npr.org/2020/05/11/854115407/tanzanias-president-blames-fake-positive-tests-in-the-spike-in-coronavirus-cases). The traditional administrative leaders – keepers of sociocultural values – had a strong desire to support any Magufuli ideas and strategies, including the approaches against COVID-19. Therefore, as long as Magufuli was strongly against modern ways of preventive measures against COVID-19, those who resisted the vaccines were also influenced by this factor.

### Medical pluralistic practices

The study found that participants believed most people in their communities were inclined towards the use of multiple medical approaches in response to illnesses, and the same was pertinent in the case of the COVID-19 pandemic. High belief and wide use of traditional medicines and remedies in moments of crisis, such as diseases, was an influencing pattern in responding to preventive measures. It was noticed that people strongly thought that as they have been responding to other diseases by adopting pluralistic medical interventions such as biomedicine, human interventions, religion, the folk sector and traditional remedies, a similar approach would play the same role during the COVID-19 pandemic.

In addition to the mistrust built around the COVID-19 pandemic, we observed in the discussions that many people still believed they could use local mechanisms to fight against this pandemic. It was clear that people were already using local herbs and concoctions to treat the severe fever, and others applied steam [*kujifukiza* in Swahili], a method they have used for ages to treat stringent flues and diseases that affect the breathing system, to face the COVID-19 pandemic. The study found that medical pluralism was a well-established framework for illness management; hence, people in Mtwara, Arusha, and Shinyanga did not need to invent anything but customise their use to respond to the pandemic as evident in the view of one participant among the local authority leaders:

‘… Therefore, even in times when someone had symptoms and signs of COVID-19, it was common for them to rely on traditional medicines and healing processes. At some point, symptoms and signs indicated by COVID-19 patients were attributed to witchcraft and bad evils [*Bhalogi* and *Mashetani*].’ (KII 42, Ward Executive Officer, WEO, Shinyanga)

It is, therefore, noteworthy to claim that responses to public health preventive measures against COVID-19 by various community members in the areas of this study were multiple, different, and not haphazard. The sociocultural environment was critical to the decisions made towards responding to various preventive measures.

## Discussion

In these findings, we observe that the influence of sociocultural practices was very significant in how people adopted public health measures against the COVID-19 pandemic. The practices appear to play a two-fold role as facilitators or hindrances towards the adoption of COVID-19 measures. Even with a lot of advocacy work by WHO, the Ministry of Health-Tanzania and other health stakeholders, people’s sociocultural background still manifests a significant role to play.^18,19^ The study further found that the most important public health preventive measures implemented to counter the rapid spread of COVID-19 were social and physical distancing, lockdowns, quarantine for those diagnosed with, exposed to, or suspected to have COVID-19, handwashing or using sanitisers, and taking COVID-19 vaccines.^3,13,14,15^ Several studies indicate that social distancing is one of the most effective public health measures that was highly recommended to avoid contracting COVID-19 at its inception before the discovery of vaccines.^20^ This study is in line with several other social and epidemiological studies, which show that social networks and social contact patterns have a major influence on the swift spread of contagious diseases, as in the case of the COVID-19 pandemic.^1,21,22^ Sociocultural practices, whose precondition is sociality and collective action, become the determining factor in whether public health measures to prevent the spread of COVID-19 are successful or not.

Other studies conducted in Tanzania and other nations such as Japan have corroborated the reality that people’s ways of behaving are much conditioned by what their social norms define as a standard way of life. For instance,^23^ in their study titled ‘*COVID-19 Knowledge, Attitudes, Practices, and Vaccination Hesitancy in Moshi, Kilimanjaro Region, Northern Tanzania*’, the authors found that while people knew COVID-19 pandemic symptoms and consequences, much of their attitudes and practices were mainly influenced by their sociocultural and traditional norms. This finding is like what was observed by this study in Mtwara, Shinyanga, and Arusha, whereby various contextual variables based on sociocultural practices mediated the way people responded to the preventive measures implemented against the COVID-19 pandemic. Sociocultural practices based on norms that give hegemonic powers to men and approve gendered decision-making in matters concerning health and other socioeconomic aspects have proven to be central to the outcomes of health interventions such as public health preventive measures against the COVID-19 pandemic. In several other studies conducted in different countries, such as Pakistan,^24^ Serbia,^21^ and China,^18,25^ sociocultural practices have proven to have an impact on the implementation of health interventions for the COVID-19 pandemic. For instance, similar to what we have observed as a challenge to social distancing and lockdown because of having extended families or congested areas at home and workplaces,^24^ also found out a similar case in Pakistan.

Our study has shown that during the outbreak of the COVID-19 pandemic, WHO, through various health institutions and on its website, communicated public health measures to be implemented to address the fast-spreading coronavirus.^3,5,6^ This was the best way to see a solution to help condense the risk of the crisis and make sure deaths were limited. However, with good intentions, the measures hurt people’s ways of life. We have found that people believe measures such as social distancing and lockdowns are endangering their traditional livelihood systems, something they are not ready to accept unless, according to them, the pandemic has to be disastrous, as pre-estimated by Western media (https://www.aljazeera.com/opinions/2020/5/7/the-problem-with-predicting-coronavirus-apocalypse-in-africa; https://edition.cnn.com/2020/06/16/africa/africa-coronavirus-cases-prevention-intl/index.html). In the same line of thought,^23^ it was shown that in Pakistan, for instance, people were also resistant to changing their behaviours and routine ways of life. The same was observed in northern Tanzania in the study by Chilongola et al.^24^ Further evidence from Tanzania indicates that the function of sociocultural practices did not only influence the implementation of public health measures for preventing the spread of the coronavirus; they have also been at the centre of other public health measures, such as undernutrition.^16,17^

## Conclusion

Our study makes a critical contribution to knowledge on the understanding of sociocultural practices responsible for the observed success or failure of the public health preventive measures implemented to curb the rapid spread of the COVID-19 pandemic in Mtwara, Arusha and Shinyanga regions in Tanzania. We conclude that, the implementation of interventions that aim at changing the behaviours of people should heed sociocultural factors informing people’s practices. We have noticed that sociocultural factors have a critical contribution to and can exert a huge amount of influence in shaping social behaviours, which in turn are responsible for determining the success and efficacy of COVID-19 public health preventive measures. It is evident that little to no attention is paid to sociocultural contexts in designing and implementing health interventions. The unique power of sociocultural practices deeply rooted in people’s everyday experiences is usually undermined, leading to the apt failure of public health measures.

The study recommends that various actors, such as international communities responsible for health, government, healthcare experts, scientists, traditional and religious leaders, civil society, all forms of media and local communities, should play their role in inhibiting any forms of misinformation by making sure the right information is shared timely and sensitisation campaigns to halt any misconceptions and rumours are provided in a timely manner. This means necessary amendments to the approaches previously applied should be performed proactively instead of waiting for another pandemic to happen. Integrating sociocultural practices into the public health measures is the ultimate responsibility for all actors.
